# Achieving 3-D Structural Uniformity in Cellulose Gel Beads via Salt Screening

**DOI:** 10.3390/polym16243519

**Published:** 2024-12-18

**Authors:** Matthew T. Garnett, Seyed Armin Seyed Esfahani, Andrew P. Yingst, Luke T. May, Symone L. M. Alexander

**Affiliations:** Department of Chemical Engineering, Auburn University, 212 Ross Hall, Auburn, AL 36849, USA; mtg0019@auburn.edu (M.T.G.);

**Keywords:** cellulose beads, self-assembly, hydrogels, microgels

## Abstract

Cellulose microgel beads fabricated using the dropping technique suffer from structural irregularity and mechanical variability. This limits their translation to biomedical applications that are sensitive to variations in material properties. Ionic salts are often uncontrolled by-products of this technique, despite the known effects of ionic salts on cellulose assembly. In this study, the coagulation behavior of cellulose/salt solutions was explored as a way to combat these challenges. An ionic salt (NaCl) was added to a cellulose solution (cellulose/NaOH/urea) prior to coagulation in a hydrochloric acid bath. Quantification of the bead geometry and characterization of the pore architecture revealed that balancing the introduction of salt with the resultant solution viscosity is more effective at reducing structural variability and diffusion limitations than other pre-gelling techniques like thermal gelation. Three-dimensional visualization of the internal pore structure of neat cellulose, thermo-gel, and salt-gel beads revealed that adding salt to the solution is the most effective way to achieve 3-D structural uniformity throughout the bead. Coupled with nanoindentation, we confirmed that the salt produced during coagulation plays a critical role in mechanical variability, and that adding salt to the solution before dropping into the coagulation bath completely screens this effect, producing uniform microgel beads with reproducible mechanical properties.

## 1. Introduction

Cellulose displays a diverse array of material properties in nature, including texture, mechanical response, and transport of water and other small molecules [1,2,3]. These unique attributes are driven by its hierarchical self-assembly, where individual cellulose chains interact and assemble across length scales to form fibers, bundles, and macroscopic materials [4,5]. Inspired by properties observed in nature, researchers have used cellulose to design and fabricate gels and composites for many applications that span fields of polymer science, engineering, and even sensor technology [4,6,7]. Cellulose is also biocompatible, biodegradable, bio-absorptive, and has low toxicity [8]. Therefore, cellulose and its derivatives have become critical matrix materials for biomedical applications such as drug delivery [8]. Specifically, oral delivery is a key area of interest because the human body does not readily digest cellulose, creating an opportunity to design drug delivery platforms that will remain stable and provide sustained drug release in the gastrointestinal tract [9].

Cellulose microgels are typically formed using the dropping technique, where a cellulose solution is extruded dropwise from a syringe to a coagulation bath that causes cellulose to self-assemble and form porous spheres called microgels or beads [10]. Cellulose with concentrations up to 2 wt% can be fully dissolved in 8 wt% sodium hydroxide/urea solutions at low temperatures (−10 °C) [11,12]. At 2 wt%, cellulose chains begin to assemble into rod-like fibrillar structures in solution [13,14,15,16,17]. In the dropping technique, the cellulose solution is dropped into a beaker containing an aqueous acid like hydrochloric acid (HCl) [10,18,19]. The resultant neutralization of sodium hydroxide (NaOH) decreases the solubility of the cellulose, leading to coagulation and microgel bead formation. If the cellulose concentration is increased, the aggregate size and the rate of coagulation also increase. After coagulation, the beads are washed, neutralized and either freeze-dried or stored in water. Even though the methodology is straightforward, the resultant material properties are complex. Numerous experimental factors can negatively impact the properties of the microgels, including dropping height, needle gauge size, the composition and temperature of the coagulation bath, and the concentration and temperature of the cellulose solution [10,20,21]. Even after optimizing these factors, cellulose microgels exhibit high variability in mechanical properties and do not survive in vitro digestion processes [22,23,24].

One way to reduce the variability of cellulose beads is via thermal gelation [10,25]. As the temperature of the cellulose solution increases, the cellulose solubility decreases, leading to gel formation. When used in conjunction with the dropping technique, thermal gelation stabilizes the cellulose beads due to increased solution viscosity and helps maximize the rate of coagulation [26]. The physical crosslinking helps limit the movement of polymer chains in the solution as the droplet is introduced to the coagulation bath [25,27]. Thermal gelation times are also dependent on the cellulose concentration; a higher concentration decreases the gelation time [10]. While thermal gelation improves geometric features like circularity, the variability of the pore structure and material properties persists. Thus, more than gel pre-assembly is needed to overcome these material design challenges.

The driving force behind the formation of cellulose beads via the dropping technique is the neutralization of the base solvent (NaOH) with an acidic bath (HCl) [19]. This neutralization causes the cellulose in the droplet to aggregate and self-assemble, leading to spherical microgels. An overlooked feature of this process is the production of salt, specifically sodium chloride (NaCl), from the neutralization reaction:

(1) $$NaOH\;\left(aq\right)+HCl\left(aq\right)\to NaCl\left(aq\right)+H_{2}O\left(l\right)$$

NaCl and other ionic salts are known to influence the hydration and assembly of cellulose, where an increase in salt concentration causes the cellulose to form a gel even in NaOH solutions [28,29]. This phenomenon is known as the “salting out” effect and is demonstrated by cellulose nanofibers (CNFs), cellulose nanocrystals (CNCs), and other biopolymers like proteins [28,29]. Recently, Arola et al. used experimental and computational techniques to reveal that ionic salts create bridges between cellulose nanofibers, allowing for stronger, more ordered contacts to form between fibrils [30]. In their study, small deformation oscillatory rheology revealed that CNF suspensions with low NaCl concentrations (0.25–1 mM) take longer to recover from stress, while cellulose suspensions with higher NaCl concentrations (above 2 mM) form highly elastic networks that quickly recover from stress [30]. Overall, Arola et al. concluded that even trace amounts of NaCl significantly influence the self-assembly of CNF and the stress response of the resultant material and play a critical role in the mechanical variability that is frequently observed in the literature [30]. CNCs suspended in solution form a heterogenous mixture where they retain their macroscopic structure and can form larger clusters due to interparticle attractions and hydrogen bonding between the charged groups on the surface of the CNC and water [31]. CNCs display higher surface area, modulus, and negative surface charge, and a rod-like crystalline structure compared to CNFs [32]. Cao et al. investigated the aggregation kinetics for CNC suspensions containing monovalent, divalent, and trivalent salts [33]. At NaCl concentrations above 153 mM, rapid assembly occurs and causes instability in the colloidal suspensions of CNCs [33].

The prior literature focuses only on CNF and CNC suspensions—rigid particles dispersed in a solvent—but the dropping technique involves a homogenous cellulose solution, which includes cellulose aggregates distributed throughout a NaOH solution. The addition of salt can then cause the dissolved cellulose to self-assemble into larger aggregates and form gel networks [28,29,34,35]. During the dropping technique, the salt concentration varies rapidly as the neutralization reaction proceeds. Thus, we hypothesize that the salt fluctuations are causing significant changes in aggregation behavior and kinetics, leading to non-uniform internal bead structures and gel networks.

In this work, we explored the addition of salt to cellulose solutions prior to dropping as a tool to tune the micro- and macrostructure of cellulose microgels. Our goal was to achieve structural uniformity and reduce variability in mechanical properties for microgels fabricated using the dropping technique. We also compared salt addition to physical methods of aggregation (thermo-gel) to decouple cellulose aggregation caused by the increase in temperature of the coagulation bath from the aggregation caused by ionic salt formation. We show that thermal gelation is not sufficient to overcome the structural irregularities caused by salt formation and that balancing solution viscosity with the addition of salt is highly effective at achieving structural uniformity and reproducible mechanical properties. Additionally, we provide a critical 3-D viewpoint of the effects of salt on the internal structure of cellulose microgel beads.

## 2. Materials and Methods

### 2.1. Materials

Fibrous cotton linter pulp cellulose, hydrochloric acid (ACS reagent, 37%), urea, sodium chloride, sodium hydroxide (ACS reagent, >97%) pellets, pepsin, potassium dihydrogen phosphate, and trypsin were purchased from Millipore Sigma (Burlington, MA, USA).

### 2.2. Fabrication of Neat (Control) Cellulose Solutions

Cotton linter pulp was dissolved in a solution of 7 wt% NaOH, 12 wt% urea, and 81 wt% water to obtain an overall solution of 5 wt% cellulose. The solution was transferred to a jacketed beaker connected to a chiller at −10 °C and allowed to stir until a transparent solution was observed. The solutions were degassed by centrifugation at −10 °C and 8000 rpm.

### 2.3. Fabrication of Salt-Gel Solutions

Salt pre-gelled (salt-gel) solutions were formed following the same procedure as the neat cellulose solutions with the following modification: NaCl (0.5 wt% (111 mM), 1 wt% (225 mM), 2 wt% (449 mM), and 3 wt% (673 mM)) was added to the solution and stirred until fully dissolved before the addition of cellulose. The solution was transferred to a jacketed beaker connected to a chiller at −10 °C and allowed to stir until a transparent solution was observed. The solutions were degassed by centrifugation at −10 °C and 8000 rpm.

### 2.4. Fabrication of Thermo-Gel Solutions

Thermally pre-gelled (thermo-gel) solutions were formed following the same procedure as the neat cellulose solutions. After degassing, the centrifuge tube was removed and placed in a water bath with the liquid level in the centrifuge tube completely submerged under the water level of the water bath. The bath temperature was set to 25 °C, 45 °C, or 65 °C. Each trial was conducted in triplicate.

### 2.5. Heat Transfer Modeling for Thermo-Gel Solutions

To minimize structural variability caused by heat transfer, Python (version 3.11) was used to predict the temperature profile within the centrifuge tube as a function of bath temperature. These computational results were used to determine the water bath temperatures used to fabricate thermo-gels and were validated experimentally. For this model, the initial temperature of the cellulose solution is set to −10 °C, and heat transfer was modeled as a function of the temperature of the water medium.

To solve the heat equation in cylindrical coordinates, the heat transfer was considered only in the radial (*r*) direction

(2) $$\rho C_{p}\frac{\partial T}{\partial t}=k\frac{1}{r}\frac{\partial }{\partial r}\left(r\frac{\partial T}{\partial r}\right)$$

where ρ is the density, $C_{p}$ is the heat capacity, ∂T/∂t is the partial derivative of temperature with respect to time, k is the thermal conductivity, r is the radius, and ∂T/∂r is the partial derivative of temperature with respect to radius [36].

The initial condition is that the temperature of the cellulose solution is −10 °C. The first boundary condition is for the outer surface of the centrifuge tube, which is in contact with the water medium, and the conductive heat flux is equal to that of water. The second boundary condition is that, at the center of the tube (r=0), the temperature gradient over time is finite.

(3) $$\left\{\begin{array}{c} I.C.:\;\;\;\;at\;t=0\;\;\;\;\;\;T\left(r,0\right)=T_{0}\\ B.C.1:\;\;\;\;at\;r=R\;\;\;\left(-k\right)\frac{\partial T\left(R,t\right)}{\partial r}=h\left[T\left(R,t\right)-T_{w}\right]\\ B.C.2:\;\;\;\;at\;r=0\;\;\;T\left(0,t\right)=finite\; \end{array}\;\right.$$

The mathematical method used to solve the heat equation was separation of variables, and the Newton–Raphson method was implemented to find the eigenvalues used to determine the temperature distribution as a function of distance from the center of the centrifuge tube (r=0) and time [37,38].

### 2.6. Microgel Formation via the Dropping Technique

**Figure 1:**
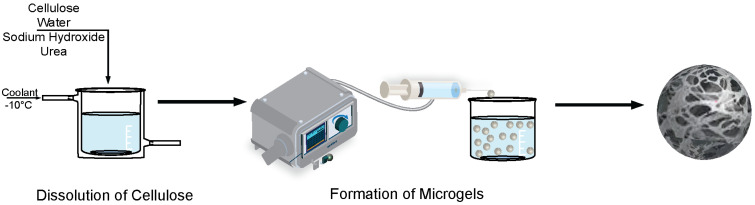
Cellulose microgel beads are formed using a dropping technique in a 2M HCl coagulation bath.

First, 5 mL of cellulose solution (neat, salt-gel, or thermo-gel) was loaded into a syringe and dropped at a flowrate of 0.2 mL/min from a horizontally oriented 30-gauge needle at a dropping height of 1 cm from a 2 M HCl coagulation bath (Figure 1). The 2 M HCl coagulation bath was held at 25 °C with constant stirring of 100 rpm to prevent the beads from settling at the bottom of the beaker and to ensure uniform concentration throughout the coagulation bath. Once all of the cellulose solution was added to the bath, the cellulose beads were continuously stirred at 100 rpm for 2 h. The beads were washed with a total of 600 mL of deionized water (200 mL, 3×. After the last wash, a 0.1 M NaOH solution was added dropwise to the DI water bath containing the rinsed beads until a neutral pH was obtained. The beads were removed from the bath and placed in a centrifuge tube with fresh DI water. The centrifuge tube was placed in an incubated shaker at 25 °C and an agitation rate of 100 rpm for 24 h to allow the microgels to equilibrate. Finally, the beads were flash-frozen with liquid nitrogen and freeze-dried using a LabConco FreeZone Freeze-Dryer.

### 2.7. Determination of Viscosity

Approximately 10 mL of cellulose/NaOH/urea solution at 20 °C was placed in a Cannon Instruments Ubbelohde viscometer, and the time taken for the solution to move from one specified point to another was recorded. A Cannon Instruments (State College, PA, USA) 150 Ubbelohde viscometer was used for the CNF solutions, while a Cannon Instruments 100 Ubbelohde viscometer was used for the CNF suspensions (DI Water and NaOH/Urea). The dynamic viscosity can be calculated using Equation (4)

(4) η=cρt

where η is the viscosity of the solution in cP, c is the Ubbelohde viscometer’s specific constant, ρ is density in g/cm^3^, and t is time in seconds [39].

### 2.8. Determination of Particle Size and Electrophoretic Mobility (EPM)

Particle size and electrophoretic mobility were measured using dynamic light scattering (DLS) via a Malvern Zetasizer Nano ZS.

### 2.9. Microgel Size and Shape Characterization

Image J (version 1.53k) was used to measure the diameter of the selected cellulose beads and calculate the volume, area, and perimeter [40]. The circularity of the beads was quantified using Equation (5):

(5) $$C=\sqrt{\frac{4\pi A}{P^{2}}}$$

where C is the circularity, A is the area, and P is the perimeter [41]. The resulting size and circularity distributions were fitted to a normal distribution and statistical analysis was used to determine statistical significance. To determine whether the diameter and circularity data were statically significant, a Shapiro–Wilke test was performed on the means to determine normality. Once normality was confirmed, a *t*-test was performed to compare the neat cellulose and thermo-gel beads for both diameter and circularity to determine whether the means were statistically significant. For the salt-gel beads with varying salt concentrations, a one-way ANOVA was performed to determine whether the means between the salt-gel beads and the neat cellulose beads were statistically significant for both diameter and circularity measurements.

### 2.10. Scanning Electron Microscopy (SEM)

The microstructure of neat cellulose, salt-gel, and thermo-gel beads were analyzed using a ThermoFisher (Waltham, MA, USA) Scientific Phenom ProX Desktop Electron Microscope with a voltage of 5 kV and a magnification of 2000× or 15,000×. Both surface and cross-sectional images were taken. The beads were sputter-coated with gold using a Q150T ES Plus Electron Microscopy Sciences Sputter Coater prior to analysis. The porosities of neat cellulose, salt-gel, and thermo-gel beads were analyzed by importing SEM images of each bead into ImageJ.

### 2.11. Energy-Dispersive X-Ray Spectroscopy (EDS)

Energy-Dispersive X-ray Spectroscopy (EDS) analysis was performed on neat cellulose and salt-gel beads using an INCA X-Stream 2 Energy-Dispersive X-ray Spectrometer in combination with a ZEISS (Jena, Germany) EVO50 Scanning Electron Microscope with a voltage of 5 kV. Neat cellulose and salt-gel beads were examined both prior to and after washing to determine the elemental make-up of each sample. Analysis was performed on both the surface and cross-section of the beads to determine if salt was present after fabrication and neutralization (Table S3).

### 2.12. Three-Dimensional X-Ray Computed Tomography (Nano-CT)

Mounted cellulose beads were mounted on toothpicks and scanned with an air filter in a ZEISS (Jena, Germany) Xradia Versa 3D X-ray Computed Tomography (CT) Scanner with a 4X objective. A total of 801 projections were taken as the sample rotated 360 degrees. To increase image quality, the binning was set to 8 and the exposure time was set to 0.5 s. A voltage of 50 kV and power of 2 W were used during the scanning process. At the conclusion of scanning, the 2D projected images were transferred to the ZEISS Scout and Scan Control System software for reconstruction. Finally, 360-degree rotations of the 3-D reconstructions were created using FIJI [42].

### 2.13. Determination of Droplet Volume

The volume extruded from a syringe and dropped into the HCl coagulation bath was determined by dropping 5 mL of cellulose solution into a 10 mL graduated cylinder. During dropping, the total number of droplets was recorded. Therefore, to determine the volume of a droplet, the overall volume collected in the graduated cylinder was divided by the total number of droplets.

### 2.14. Simulated Gastrointestinal Tract Environment for Cellulose Beads

Cellulose beads were tested in a simulated gastrointestinal tract environment to understand the swelling and mechanical properties of the beads. Simulated gastric fluid (SGF) was prepared by dissolving 4.5 g of sodium chloride and 1.6 g of pepsin in 500 mL of water [24]. Simulated intestinal fluid (SIF) was prepared by dissolving 3.4 g of potassium dihydrogen phosphate and 5 g of trypsin in 500 mL of water [24]. Cellulose beads were placed in SGF incubated at 37 °C for 2 h with an 80 rpm agitation. At the conclusion of the 2 h, the beads were removed from the SGF and then placed in SIF for 6 h at 37 °C with an 80 rpm agitation [37,38].

### 2.15. Mechanical Testing

The mechanical properties of neat cellulose, salt-gel, and thermo-gel beads were measured using the FEMTO Tools^®^ Micromechanical Testing and Assembly System (FemtoTools, Buchs, Switzerland) after undergoing the simulated gastrointestinal tract. To mechanically test the beads in the simulated gastrointestinal tract, 15 cellulose beads were first placed in SGF incubated at 37 °C for 2 h with an 80 rpm agitation. At the conclusion of the two hours, the beads were tested via nanoindentation. Then, the beads were incubated in SIF for 6 h at 37 °C with an 80 rpm agitation prior to undergoing nanoindentation. The Young’s modulus was determined using nanoindentation with a spherical ruby-tip probe. Force versus displacement curves were fit to the Oliver–Pharr model to determine the Young’s modulus [43]. The equations used to determine the Young’s modulus can be found in the Supplemental Materials. To determine whether the data were statically significant, a Shapiro–Wilke test was performed by means of the Young’s moduli to determine the normality. Once normality was confirmed, a one-way ANOVA was performed to determine statistical significance between water, SGF, and SIF for a particular bead type. A two-way ANOVA was performed to determine whether the means between each bead type in water, SGF, and SIF were statistically significant.

### 2.16. Swelling Experiments for Cellulose Beads in a Simulated Gastrointestinal Tract Environment

Prior to incubating beads in SGF and SIF, images of fifteen neat cellulose, thermo-gel, and salt-gel beads were taken and analyzed using ImageJ to determine the volume. The beads were then incubated in SGF at 37 °C for two hours with an 80 rpm agitation. At the completion of two hours, the beads were removed, and images were taken to determine the volume of the beads using ImageJ. After imaging, the beads were placed in SIF and incubated for six hours at 37 °C with an 80 rpm agitation. At the conclusion of six hours, the beads were removed, and images were taken to determine the volume. Using the initial and final volumes of the beads measured using Image J, the volume–swelling ratio was determined using Equation (6)

(6) $$Q=\frac{V_{final}}{V_{initial}}$$

where Q is the volume–swelling ratio, $V_{initial}$ is the initial volume before subjecting the beads to the simulated gastrointestinal tract, and $V_{final}$ is the final volume after incubation [44,45].

## 3. Results and Discussion

### 3.1. Effect of Thermal Gelation on Cellulose Solutions

Thermal gelation was used to decouple the effects of added salt from the effects of the increased temperature of the coagulation bath. Thermally driven gelation for a cellulose/urea/NaOH solution occurs between 10 and 65 °C [21]. For this study, a model was used to predict the temperature distribution within the cellulose solution contained in a centrifuge tube. This computational approach helped to determine an ideal bath temperature that would minimize thermal gradients and reduce variability in assembly due to thermal fluctuations. The computational results were compared to experimental measurements of solution temperature vs. time at different bath temperatures (25, 45, and 65 °C). The minimum water bath temperature was set to the temperature of the HCl coagulation bath (25 °C) that would be used to form the microgel beads. The initial temperature of the cellulose solution was set to −10 °C to match the experimental solution fabrication conditions. The heat transfer was modeled within the cylindrical coordinate system as a function of radius (r) and time.

**Figure 2:**
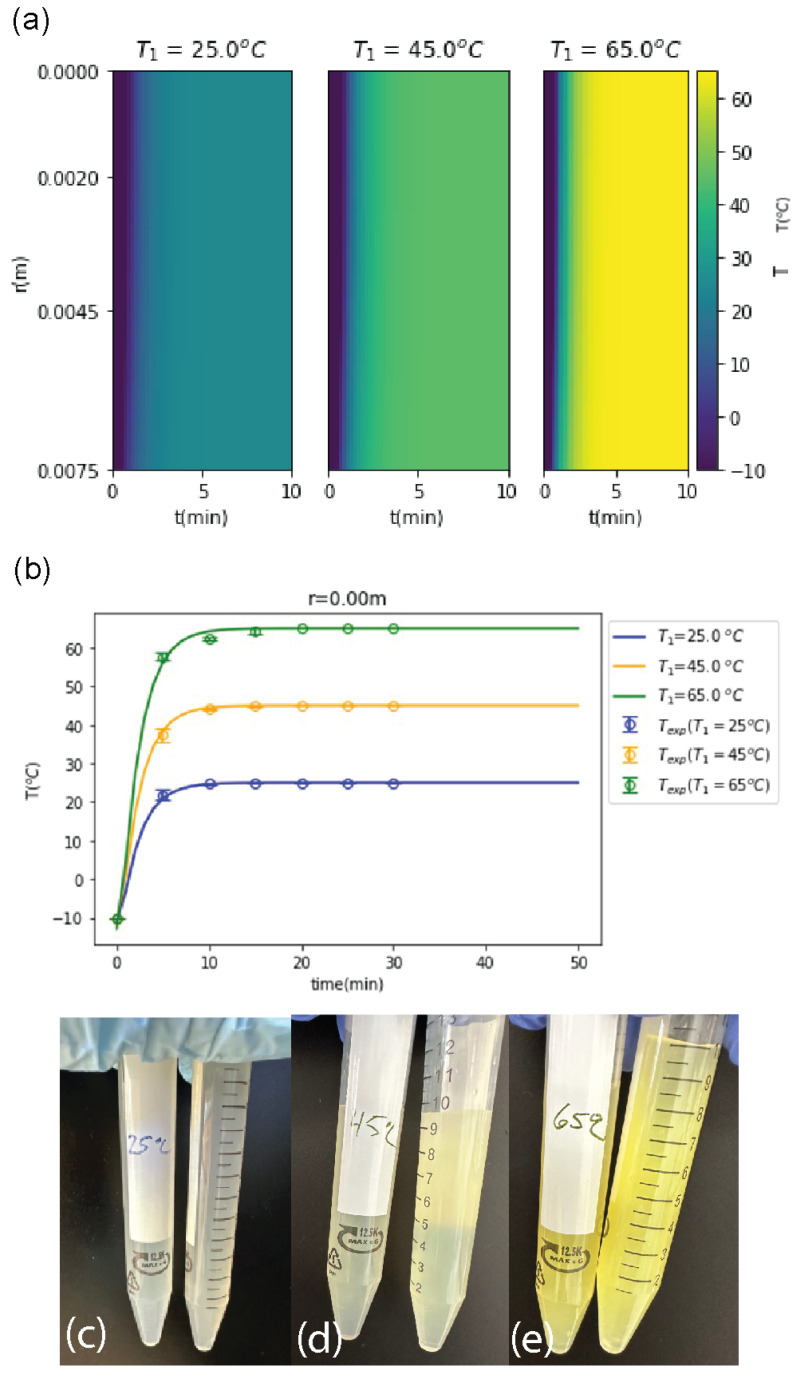
(**a**) Computational heat maps were used to predict thermal gradients present in cellulose solutions over time as a function of bath temperature. A 25 °C water bath resulted in the lowest thermal gradient as a function of radial distance and time, indicating the least amount of thermal stress. (**b**) Experimental temperature vs. time data were used to validate the model (r = 0.00 m) and determine the time required to reach thermal equilibrium for water bath temperatures of 25, 45, and 65 °C. (**c**–**e**) Solutions were visually examined for signs of thermal stress (transition from colorless to yellow) and precipitation (transition from transparent to opaque). Signs of thermal stress increased as a function of bath temperature, with the 25 °C water bath (**c**) showing the least signs of thermal stress after 30 min compared to the 45 °C (**d**) and 65 °C (**e**) water baths.

Heat maps were used to visualize the radial temperature variation from the center (r = 0 m) to the surface (r = 0.0075 m) of the tube over time for each water bath temperature (Figure 2a). The heat map employs a color gradient from blue to yellow as a function of the temperature for each radial position over time. For all bath temperatures, the initial temperature of cellulose solution at the center of the tube (r = 0) is −10 °C. As expected, a radial temperature gradient was observed and reached its maximum at the surface of the tube (r = 0.0075 m), where the temperature matched that of the surrounding water bath. As time progressed, the temperature gradient between each radial point decreased. Notably, the cellulose solution in the 25 °C bath displayed lower thermal gradients as a function of radial distance and reached thermal equilibrium faster than the 45 °C, and 65 °C baths, with all bath temperatures reaching thermal equilibrium within 5-10 min. This indicates that a 25 °C bath is less likely to cause thermal stress or variations in cellulose assembly and will potentially produce a more uniform thermo-gel solution.

The computational model was validated with experimental data, as shown in Figure 2b). Sealed centrifuge tubes (15 mL, r = 0.0075 m) containing −10 °C neat cellulose solutions were placed in a water bath with a set temperature of 25, 45, or 65 °C. The temperature of the cellulose solution was measured over time. As shown in Figure 2b), most of the heat transfer in the centrifuge tube occurred within the first five minutes. After ten minutes in the water bath, all the cellulose solutions began to approach thermal equilibrium, which is consistent with the model’s predictions.

After 30 min, the centrifuge tubes were removed from the water bath and visually inspected for changes in color or transparency (Figure 2c). Changes in color, particularly to yellow, indicate decomposition and denaturation from chemical, photo, or thermal stress [46,47]. Changes in transparency indicate precipitation of cellulose from solution. The neat cellulose solution is transparent and colorless at −10 °C. The cellulose solution that was placed in the 25 °C bath remained transparent, but slight yellowing of the solution was observed after 30 min. For the 45 °C and 65 °C bath temperatures, the cellulose solutions displayed a distinct yellow tint, and the 65 °C system became significantly less transparent. For the 45 °C and 65 °C bath temperatures, the cellulose solutions displayed a distinct yellow tint, and the 65 °C system became significantly less transparent. Therefore, higher bath temperatures cause decomposition and/or precipitation of cellulose from solution and are not feasible for forming thermo-gels. Even the 25 °C system exhibited signs of thermal stress, indicating that using salt could be an advantageous way to prevent damage to the cellulose solution when using a pre-gelling approach to form uniform microgels.

### 3.2. Effect of Added Salt on Cellulose Solutions

Dropping a dissolved CNF/NaOH/Urea/Water solution into an HCl coagulation bath naturally makes saltwater via the previously described neutralization reaction between NaOH and HCl (Equation (1)). By calculating the volume of an individual droplet, we determined that the NaCl concentration produced in a single droplet is ~2100 mM. Thus, when droplets of the cellulose solution are added to the coagulation bath, the neutralization reaction causes a significant increase in the salt concentration in the droplet. The rapid increase in salt concentration then causes rapid flocculation and significant variability in self-assembly and microstructure and as it forms, the droplet develops into a microgel. To counteract this effect, different salt concentrations (0.5, 1, 2, and 3 wt%) were added to neat cellulose solutions prior to dropping in the coagulation bath. The weight fractions represent salt concentrations of 112, 225, 449, and 673 mM, respectively.

**Figure 3:**
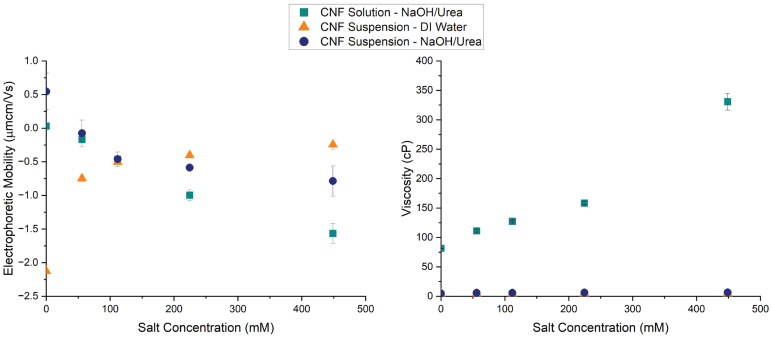
Electrophoretic mobility and dynamic viscosity were compared for a CNF solution and CNF suspensions. The presence of NaOH in solution leads to opposite trends for CNF suspensions. In DI water, the CNFs display a net negative charge that decreases as the salt concentration increases. In a NaOH/Urea solution, the CNFs display a net positive charge that becomes more negative as the salt concentration increases. Dissolved CNFs in a NaOH solution displayed an electrophoretic mobility near zero that became more negative as the salt concentration increased. Both of the CNF suspensions displayed negligible changes in viscosity as the salt concentration increased compared to the CNF solution. The electrophoretic mobility and viscosity of the suspensions and solutions were measured at 20 °C.

We quantified and compared the electrophoretic mobility and the dynamic viscosity as a function of salt concentration for CNF suspensions (CNF suspended in DI water and NaOH/Urea) and a CNF solution (CNF dissolved in a NaOH/Urea aqueous solution) (Figure 3 and Table S1). Electrophoretic mobility is a measure of the migration velocity of an ion or molecule in a solution under an electric field [48,49]. The sign and magnitude of the electrophoretic mobility indicates the net charge on the molecule or ionic species. The electrophoretic mobility of the CNF suspension in DI water became less negative as the salt concentration increased. CNFs display a negative surface charge due to the presence of both hydroxyl and carboxyl groups [50,51]. When the salt concentration is increased, the positive Na^+^ ions surround and screen the negatively charged groups of cellulose, which in turn neutralizes their charge and decreases their electrophoretic mobility [52,53]. For the CNF suspension in the NaOH/Urea solution, the electrophoretic mobility is initially positive, likely due to electrostatic interactions between cellulose and NaOH, then becomes more negative as the salt concentration increases. The “common ion effect” could explain the displacement of ions from cellulose molecules, whereby excess sodium (Na^+^) ions introduced to the solution by adding NaCl disrupts the electrostatic interactions between cellulose molecule and the pre-existing Na^+^ and OH^-^ ions [54]. The CNF solution in NaOH/Urea showed a similar trend, where the electrophoretic mobility became more negative as the salt concentration increased. Therefore, for both the CNF solution and CNF suspension in NaOH/urea, the increase in salt concentration causes the cellulose to become more negatively charged and increases the electrophoretic mobility [55]. Additionally, water has strong interactions with salt but tenuous interactions with cellulose, and the higher concentrations of salt could cause dewetting of the cellulose in preference for salt [30]. In all solutions, increasing the salt concentration increased the hydrodynamic radius (Table S2).

In terms of viscosity, as the salt concentration increased, the viscosity of the CNF solution increased. In contrast, as the salt concentration of the CNF suspensions increased, the viscosity displayed negligible changes. We hypothesized that the increase in viscosity of the CNF solution as a function of salt concentration would be beneficial for bead formation. If the salt concentration is high enough in the droplet, the cellulose molecules can form a uniform, elastic network that can quickly recover from the shear forces of being extruded from the syringe into the coagulation bath (decreasing tail formation). As the salt concentration increases, the viscosity and elasticity of the salt-gel solution increases to an upper bound where the solution becomes a rigid gel that can no longer be extruded through the syringe [30,56]. The addition of salt also allows for larger channels to form in the hydrogel network and increased water transport compared to hydrogels with lower concentrations of salt or solutions without salt [52]. All of the salt concentrations except for 3 wt% formed solutions that could still be extruded through the syringe. Thus, we proceeded to form microgels from neat cellulose solutions, thermo-gel solutions, and salt-gel solutions with salt concentrations of 112, 225, and 449 mM, or 0.5, 1, and 2 wt%, respectively.

### 3.3. Effect of Thermal and Salt Gelation on Cellulose Bead Geometry

**Figure 4:**
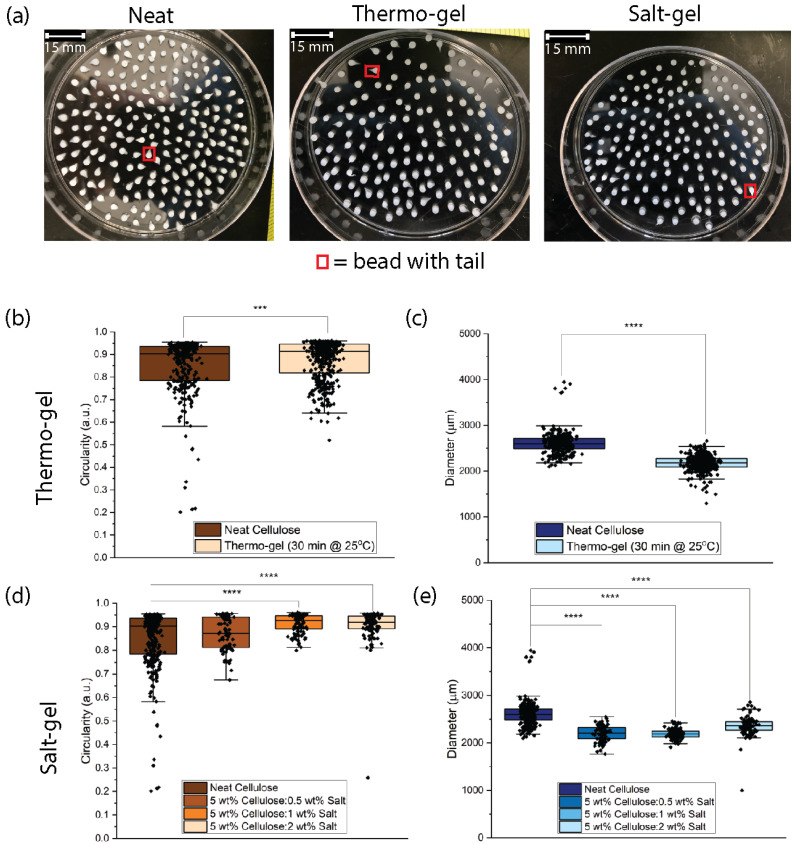
(**a**) Neat cellulose, thermo-gel, and salt-gel beads were examined for tail formation using ImageJ. The red square indicates a bead with tail formation. Salt-gel beads had the lowest degree of tail formation compared to neat cellulose and thermo-gel beads. (**b**,**c**) The circularity of the thermo-gel beads slightly increased compared to that of the neat cellulose beads and had lower variability. The diameter of the thermo-gel beads was smaller than that of the neat cellulose beads. (**d**,**e**) Salt-gel beads with a salt concentration of 0.5 wt% did not improve bead geometry, while 1 and 2 wt% salt increased circularity and decreased variability compared to both neat cellulose and thermo-gel beads. A t-test was used to determine if there were significant differences between the diameter and circularity of neat and thermo-gel cellulose beads. A one-way ANOVA was used to determine if the means of the diameter and circularity were significantly different between the neat and salt-gel beads. *** *p* < 0.001, **** *p* < 0.0001, and no * = not significant.

Neat cellulose control beads were fabricated using the dropping technique. Overall, 42% of the neat cellulose beads displayed tail formation, where the coagulated beads have a teardrop geometry rather than a spherical geometry (Figure 4a and Figure S1). ImageJ was used to determine the diameter and circularity from images and used to quantify differences in bead geometry. Neat cellulose beads had an average diameter of 2.60 ± 0.24 mm and a circularity of 0.80 ± 0.10, where perfect spheres would have a circularity value of 1 (Figure 4). In previous work, neat cellulose beads had an average diameter of ~2–3 mm and ranged in circularity from ~0.68 to 0.90 [10]. However, no one has quantified the percentage of beads displaying tail formation. Instead, they correlated the change in circularity to fewer beads with tails being formed [10,57,58]. To increase circularity and decrease tail formation, the coagulation bath properties, such as temperature and concentration, and dropping height were adjusted. This work presents a new approach, screening ionic salt-induced aggregation in the cellulose solution, to improve bead size, shape, and reduce tail formation.

Thermo-gel solutions were fabricated by placing the degassed cellulose solutions in a 25 °C water bath for 30 min and immediately extruding the solution dropwise into a 2 M HCl coagulation bath. The percentage of tail formation for thermo-gel cellulose beads decreased to 27%, compared to the neat cellulose solution (Figure 4a). Thermo-gel cellulose beads had an average diameter of 2.19 ± 0.06 mm and a circularity of 0.88 ± 0.02 (Figure 4b,c). Thus, the thermo-gel cellulose beads had a lower diameter than the neat cellulose beads and only a slight increase in circularity.

The 0.5 wt% salt-gel beads displayed similar results to the neat cellulose beads, where 43% of the beads exhibited tail formation and the diameter and circularity values were 2.20 ± 0.17 mm and 0.87 ± 0.07, respectively. Cellulose solutions formed using lower salt concentrations have weaker contact between cellulose fibers and are not able to recover from deformation or shear like the cellulose solutions formed using higher salt concentrations [30]. Therefore, the data indicate that a salt concentration of 0.5 wt% does not provide the solution elasticity needed to decrease tail formation.

Adding 1 wt% NaCl into the cellulose solution decreased the number of tails observed in the beads to 18%. A higher circularity (0.91 ± 0.04) and smaller bead diameter (2.19 ± 0.10 mm) were also observed. Salt-gel solutions with higher salt concentrations can form more spherical beads due to the network elasticity imparted by the percolated network of salt bridges that improve the contact strength of the cellulose fibers. This enables the droplet to quickly recover from the shear forces of the syringe needle before coagulation locks in the teardrop structure, effectively reducing tail formation. The 2 wt% salt-gel beads provided better results compared to the neat cellulose beads and 0.5 wt% salt-gel beads (diameter = 2.36 ± 0.22 mm, circularity = 0.91 ± 0.07). However, the 2 wt% salt-gel beads had higher variability in diameter than the 1 wt% salt-gel beads, with no significant improvements to the circularity. Additionally, the 2 wt% salt beads had more tail formation (23% compared to 18%). At 2 wt%, the solution may be oversaturated with salt, leading to concentration gradients in the gel network that would increase variability [30]. The 1 wt% and 2 wt% salt-gel beads had a concentration of 225 and 449 mM, respectively, and had higher viscosities than the 0.5 wt% gel. At these higher NaCl concentrations, the rapid aggregation occurs and the viscosity of the solution is beneficial to bead formation. When comparing the salt-gel beads to the thermo-gel beads, salt-gel beads had less variability in both the circularity and diameter. Salt-gel beads also had higher circularity values and lower diameter values. Thermo-gel beads exhibit increases in viscosity due to thermal gelation, but they lack the salt concentration needed to screen rapid aggregation. Thus, both salt-screening and solution viscosity are important parameters to reduce variability for the dropping technique and 1 wt% solution was determined to be the optimal salt concentration to add to the neat cellulose solutions to reduce tail formation and decrease variability in diameter (Figure S1).

### 3.4. Effect of Thermal and Salt Gelation on the 3-D Structure

**Figure 5:**
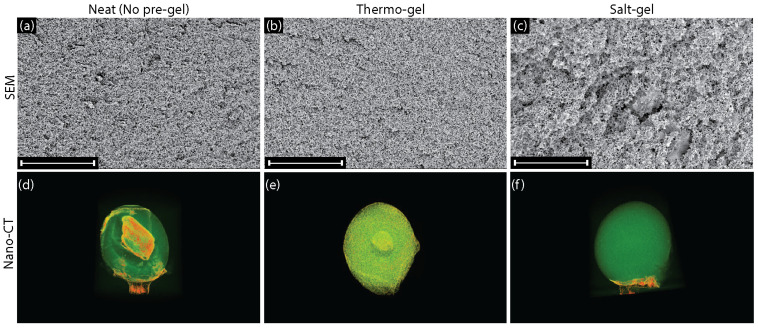
(**a**–**c**) Cross-sectional SEM images were taken of (**a**) neat (no pre-gel), (**b**) thermo-gel, and (**c**) salt-gel beads. SEM revealed that neat and thermo-gel beads had a tighter cellulose network that limited the diffusion of salt, while salt-gel beads had a larger network that allowed salt to diffuse and improve uniformity throughout the network. Neat cellulose and thermo-gel beads displayed a porosity of 54.62% and 53.09%, while the salt-gel beads displayed a porosity of 75.80%. All scale bars = 80 µm, corresponding to 2000X magnification. (**d**–**f**) 3-D nano-CT scans were taken of (**d**) neat (no pre-gel) cellulose, (**e**) thermo-gel, and (**f**) salt-gel beads fabricated in a 2M HCl coagulation bath. Nano-CT revealed that adding salt into the solution generated a uniform network structure throughout the bead. Access to 360° rotations for each bead are available in the Supplementary Information. All scale bars = 500 µm.

Scanning electron microscopy (SEM) was used to examine the internal pore structure of the microgels (Figure 5a–f). SEM images of the surface pore structure and high-magnification images of the cross-section (15,000×) are provided in Figures S2 and S3. For the neat cellulose and thermo-gel beads, a smaller pore architecture was observed. Analysis of the microstructure of the beads revealed that neat cellulose beads displayed a porosity of 54.62% and the thermo-gel beads displayed a porosity of 53.09%. In comparison, salt-gel beads displayed a larger pore architecture with a porosity of 75.80%, which indicates an easier diffusion pathway through the cellulose network. This is expected due to the restructuring and expansion of the hydration layers on cellulose fiber networks caused by the salt ions [30]. Techniques like SEM can provide visualization of the pore structure, but it is difficult to completely capture the internal structure of the bead due to the limited field of view. Therefore, we sought to use a non-destructive 3-D imaging technique to visualize the interior of the beads post-fabrication.

X-ray nano-computed tomography (nano-CT) is a 3-D X-ray imaging technique that can non-destructively scan an object and use the density distribution to visualize internal structures. Each scan creates an image slice that can be reconstructed to show the 3-D object without damaging the sample, providing a unique opportunity to visualize undisturbed internal architectures. Beads fabricated from neat cellulose, thermo-gel, and salt-gel solutions were stained, flash-frozen with liquid nitrogen and freeze-dried, then imaged via nano-CT to visualize their internal structure (Figure 5d–f). Three-dimensional reconstruction of the nano-CT scans showed that the neat cellulose beads displayed non-uniformity (Figure 5d). Throughout the bead, there are non-uniform clusters of cellulose fibers along with significant voids caused by the disconnectivity of the fiber assemblies. Additionally, the center of the bead appears to be highly ordered and much more densely packed than the surrounding shell. We hypothesize that, upon entering the coagulation bath, an initial shell is formed with the pore architecture shown in Figure 5a. Following the formation of the shell, diffusion slows due to the formation of the gel network, causing increases in local salt concentrations as the neutralization reaction proceeds. This effect would have the most drastic consequences at the center of the bead, where the NaCl produced would be present in the highest concentration due to slow diffusion from the center of the bead back through the shell.

This hypothesis is further supported by the 3-D reconstruction of the thermo-gel beads (Figure 5e). Thermo-gel beads display more structural uniformity, with a clearly interconnected gel network throughout most of the bead. However, thermo-gel beads are still subject to the salt fluctuations caused by the neutralization reaction. Though smaller in scale than the neat cellulose bead, the center of the thermo-gel bead shows a clear change in structure. As with the neat cellulose beads, the diffusion limitations are much higher at the center of the bead, leading to higher salt concentrations and changes in the assembly and microstructure. Therefore, thermally gelling the cellulose solution can improve uniformity only to the extent that it can overcome the influence of salt produced by the neutralization reaction.

**Figure 6:**
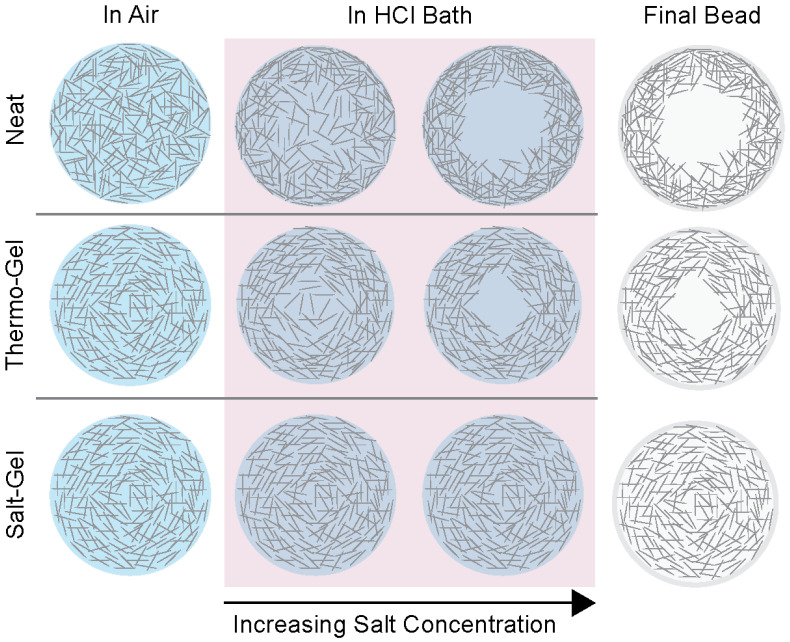
Increasing the concentration of salt into solution prior to coagulation effectively screens the salt produced via the neutralization reaction and provides elasticity to the cellulose network. Neat and thermo-gel solutions have salt concentrations that cause competition between cellulose chains during aggregation due to the lack of salt screening effects. In contrast, introducing salt into solution helps avoid this problem and leads to more uniform beads with less structural variability.

Elemental analysis of the surface and cross-section of salt-gel beads confirmed that no salt remained in the beads after neutralization (Table S3 and Figure S4). Excitingly, the 1 wt% salt-gel beads showed uniformity throughout the 3-D reconstruction (Figure 5f). A highly uniform bead was produced with no observable changes in the fiber network architecture. We concluded that adding NaCl to the cellulose solution prior to coagulation increases the elasticity of the cellulose network and successfully screens the effects of the salt produced via the neutralization reaction (Figure 6). A balance of solution viscosity and salt-screening prevents the non-uniform aggregation and coagulation of the dissolved cellulose. Ultimately, this helps reduce the variability in structure from the molecular scale to the macroscale caused by the fluctuations in salt concentration from the dropping technique. This phenomenon is also evidenced by Figure 5d,e in terms of variability in circularity and diameter for cellulose solutions with varying salt concentrations and in the nano-CT of 0.5 wt% and 2 wt% beads (Figure S4). As seen in Figure S5a,b, the 0.5 wt% salt-gel beads displayed internal structures that varied from uniform to non-uniform with tails, explaining the higher variability in the bead geometry compared to the other salt-gel beads. However, when the salt concentration was increased using 1 or 2 wt% salt-gel beads, the variability was significantly decreased. The 2 wt% salt-gel beads show uniformity throughout; however, the internal structure is much more dense, indicating a more rigid bead.

### 3.5. Proof of Concept: Mechanical Stability and Swelling Properties of Cellulose Beads in a Simulated Gastrointestinal Tract (SIT)

**Figure 7:**
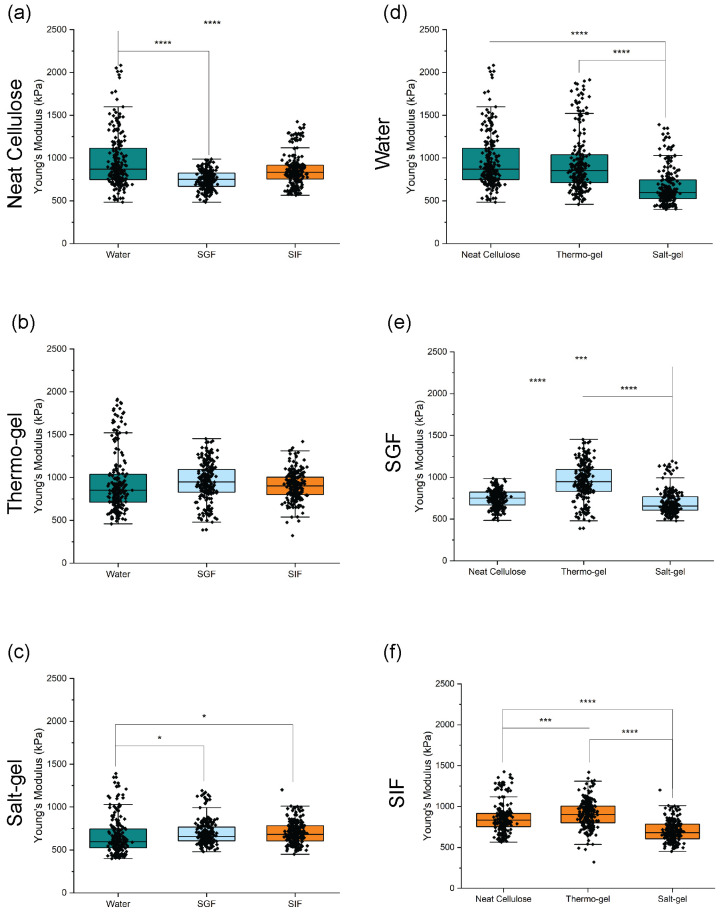
Nanoindentation was performed on (**a**) neat cellulose, (**b**) thermo-gel, and (**c**) salt-gel beads in a control (water), simulated gastric fluid (SGF), and simulated intestinal fluid (SIF) environment to compare the changes in Young’s moduli. The Young’s moduli of the neat cellulose, thermo-gel, and salt-gel beads were also compared to see if there were significant differences in the beads when subjected to (**d**) water, (**e**) SGF, and (**f**) SIF. Overall, salt-gel beads displayed lowest variability and lowest Young’s modulus compared to both thermo-gel and neat cellulose beads. (**a**–**c**) A one-way ANOVA was used to determine if the means of the three groups tested were significantly different. (**d**–**f**) A two-way ANOVA was used to determine if the means of the bead type and environment were statically significant. * *p* < 0.05, *** *p* < 0.001, **** *p* < 0.0001, and no * = not significant.

Cellulose beads are of significant interest for use in drug delivery applications due to their nondigestible properties in the gastrointestinal tract (GIT), making them a promising material platform for sustained drug release [9]. As proof of concept for the improvements to the cellulose bead material properties for GIT applications through the addition of salt, mechanical testing and swelling studies were performed in simulated GIT fluids. The mechanical properties of hydrated neat cellulose, thermo-gel, and salt-gel beads were examined to portray the effects of the 3-D architecture on mechanical variability. Nanoindentation was performed on hydrated, as fabricated cellulose beads and force vs. displacement data were fit to the Oliver–Pharr model using MATLAB^®^ (version R2023b) to determine the Young’s modulus (Figure 7). As proof of concept for the use of these cellulose beads for oral drug delivery applications, the beads were tested in a control (water) and a simulated gastrointestinal tract environment (SGF and SIF). The neat cellulose beads displayed the highest variability in mechanics with an average Young’s modulus of 966 ± 318 kPa in the control environment. As the beads underwent the simulated gastrointestinal tract, there was a statistically significant decrease in mechanics with a Young’s modulus of 747 ± 106 kPa in the SGF and 861 ± 188 kPa in the SIF. This result is comparable to mechanical testing results for cellulose beads reported in the literature (Young’s modulus = 450 kPa) [22]. The thermo-gel beads displayed a similar Young’s modulus of 941 ± 364 kPa in the control environment but, unlike the neat cellulose beads, retained similar Young’s moduli values in the simulated gastrointestinal tract with a Young’s modulus of 952 ± 224 kPa in SGF and 906 ± 165 kPa in SIF. The salt-gel cellulose beads displayed significantly lower variability in Young’s moduli, with values of 662 ± 213 kPa in the control environment, 699 ± 142 kPa in the SGF environment, and 700 ± 127 kPa in the SIF environment, respectively. Coupled with the 3-D reconstruction data, we can confirm that the notorious variations in mechanical properties exhibited by cellulose beads fabricated using the dropping technique are largely due to the structural variability caused by the salt fluctuations produced during coagulation. As seen in the control environment (water), neat cellulose and thermo-gel beads displayed similar results with high variability. On the other hand, salt-gel beads displayed lower Young’s moduli as well as lower variability; therefore, adding salt to the cellulose solution is a simple but very effective way to reduce variability by increasing structural uniformity and network elasticity throughout the solution before coagulation. The uniformity and elasticity, coupled with the screening of salt fluctuations, ultimately controls the macro- and microstructure of the coagulated beads (i.e., size, tails, and porosity) leading to reproducible mechanical properties. Even though salt-gel beads displayed the least variability in mechanical properties, statistically significant variability was present. In future work, the dropping procedure will be optimized using various salt and bath concentrations to better tune the coagulation process and further decrease variability.

In addition to mechanical testing, swelling studies were performed on neat cellulose, thermo-gel, and salt-gel beads in water (control), SGF, and SIF at 37 °C to examine the effect of the salt concentration on bead size and shrinking/swelling in different environments (Table S4 and Figure S6). All three bead platforms incubated in SGF displayed shrinkage after 2 h (0.77 ± 0.52 (neat cellulose), 0.93 ± 0.50 (thermo-gel), 0.77 ± 0.16 (salt-gel)). The observed shrinkage in SGF agrees with values previously reported in the literature for cellulose nanofiber beads that have not undergone surface modification and can be explained by weak electrostatic interactions on the surface, as well as the introduction of hydrogen bonds between the carboxyl groups [24,59]. After being incubated in SGF for two hours, the beads were placed in SIF for 6 h. All three bead platforms displayed swelling in the SIF environment compared to the SGF environment. Neat cellulose beads had a swelling ratio of 1.08 ± 0.27, compared to thermo-gel beads at 1.02 ± 0.68 and salt-gel beads at 1.05 ± 0.11. The observed swelling in SIF also agrees with previously reported values [24]. Swelling in SIF is attributed to the presence of large electrostatic repulsive forces that break the hydrogen bonds found in the beads [24,59]. Based on the swelling studies, neat and thermo-gel beads exhibit higher variability compared to the salt-gel beads. In terms of drug delivery applications, structural and mechanical uniformity are important features that influence mass transport properties and diffusion [60]. For delivery to the GIT, the beads must be able to release the drug in a desired GIT environment. For example, if the treatment needs to be delivered to the intestines, the beads need to be able to survive both the gastric tract (pH~1.5) and the intestinal tract (pH~7.5) without releasing the loaded drug prematurely and without the material degrading. The salt-gel beads would be more beneficial in this application due to their shrinkage in the intestinal fluid with lower variability than the neat cellulose beads, and their expansion in the intestinal tract to facilitate diffusion—and in light of their lower variability compared to the neat cellulose and thermo-gel beads. In the future, drug loading and release studies will be used to examine the advantages of a uniform 3-D architecture beyond reducing mechanical variability. The scientific relevance of examining diffusion through the beads also extends beyond drug delivery to other applications such as water treatment and remediation [61,62].

## 4. Conclusions

The dropping technique often used to form cellulose microgels causes high concentrations of residual salt to form due to the neutralization reaction of NaOH and HCl. The residual salt leads to structural variability in the microgel beads during coagulation, causing significant variability in mechanical properties. Previously used thermal gelation techniques provide some benefit to structural uniformity, due to their increased solution elasticity, but are sensitive to thermal stress and the production of residual salt during coagulation.

Adding salt to neat cellulose solutions effectively improves structural uniformity and drastically reduces variability in mechanical properties compared to neat cellulose solutions and thermo-gel solutions. Specifically, NaCl creates elastic, self-assembled cellulose networks in solution with larger pore architectures that easily recover from shear stress and reduce diffusion limitations during coagulation. Additionally, adding 1 wt% NaCl in the salt-gel solution effectively screens the aggregation effects of the salt produced by the neutralization reaction. The screening effect is a function of NaCl concentration, but the overall improvement to 3-D uniformity is a balance between salt-screening and solution viscosity. Overall, adding salt to cellulose/NaOH solutions is a facile and effective tool to reduce variability in structural and mechanical properties of cellulose microgel beads formed using the dropping technique.

## Data Availability

The data supporting this article have been included as part of the Supplementary Information.

## Supplementary Materials

The following supporting information can be downloaded at https://www.mdpi.com/article/10.3390/polym16243519/s1, Figure S1: Optical images of freeze-dried (a) neat, (b) thermo-gel, and (c) salt-gel cellulose beads. All scale bars = 1000 μm; Figure S2: Surface SEM images of (a) neat cellulose (no pre-gel), (b) thermo-gel, and (c) salt-gel beads. SEM revealed that neat and thermo-gel beads had smaller pore architecture that limited diffusion, while salt-gel beads had a larger pore architecture that facilitated diffusion. All scale bars = 80 μm corresponding to 2000× magnification; Figure S3: Cross-section SEM images of (a) neat cellulose (no pre-gel), (b) thermo-gel, and (c) salt-gel beads. SEM revealed that neat and thermo-gel beads had smaller pore architecture that limited diffusion, while salt-gel beads had a larger pore architecture that facilitated diffusion. All scale bars = 10 μm corresponding to 15,000× magnification; Figure S4: EDS Analysis on neat cellulose, thermo-gel, and salt-gel beads shows little to no change in percent oxygen as the HCl coagulation bath increases from 0.5 to 2 M. Salt-gel beads displayed the lowest percentage of oxygen in each of the different concentrated HCl coagulation baths; Figure S5: 3-D nano-CT scans were taken of (a,b) 0.5 wt% salt-gel and 2 wt% salt-gel beads fabricated in a 2M HCl coagulation bath. Nano-CT revealed that increasing the salt concentration improved the uniformity of the internal structure of cellulose beads. (a,b) 0.5 wt% salt-gel beads displayed both beads with a highly ordered center and uniformity throughout. (c,d) 2 wt% salt-gel beads displayed uniformity throughout; Figure S6: Swelling/Shrinkage Study of Cellulose Beads in a Simulated Gastrointestinal Tract Environment—Neat, thermo-gel, and salt-gel beads were subjected to a control (water), simulated gastric (SGF), and simulated intestinal (SIF) fluid environment and incubated for 2 h (SGF) or 8 h (SIF). The swelling ratio was calculated by the quotient of the final volume by the initial volume Table S1: The electrophoretic mobility and dynamic viscosity were measured for CNF solutions and suspensions as a function of salt concentration; Table S2: The hydrodynamic radius was measured via dynamic light scattering (DLS) as a function of salt concentration; Table S3: EDS Analysis on neat cellulose and salt-gel beads shows that the neutralization procedure washes away the salt, which is a desired result for uses of salt-gel beads for biomedical applications such as oral drug delivery; Table S4: Swelling/Shrinking Study of Cellulose Beads in a Simulated Gastrointestinal Tract Environment. A supporting information document accompanies this manuscript with additional experimental details, materials, and methods, including microscopy and tabulated values used for graphs and calculations. Reference [63] is cited in the Supplementary Materials.
