# Supplementary material for: Achieving 3-D Structural Uniformity in Cellulose Gel Beads via Salt Screening
Source: Polymers (Basel). 2024 Dec 18;16(24):3519. doi: 10.3390/polym16243519 (PMC11677921; doi:10.3390/polym16243519)
Supplement: Supplementary file 1 [file polymers-16-03519-s001.zip › polymers-3350596-supplementary.pdf]

polymers

MDPI

Supplementary Material

Achieving 3-D Structural Uniformity in Cellulose Gel Beads via Salt Screening

Matthew T. Garnett, Seyed Armin Seyed Esfahani, Andrew P. Yingst, Luke T. May and Symone L. M. Alexander *

Department of Chemical Engineering, Auburn University, 212 Ross Hall, Auburn, AL 36849, USA; mtg0019@auburn.edu (M.T.G.)
* Correspondence: sla0044@auburn.edu

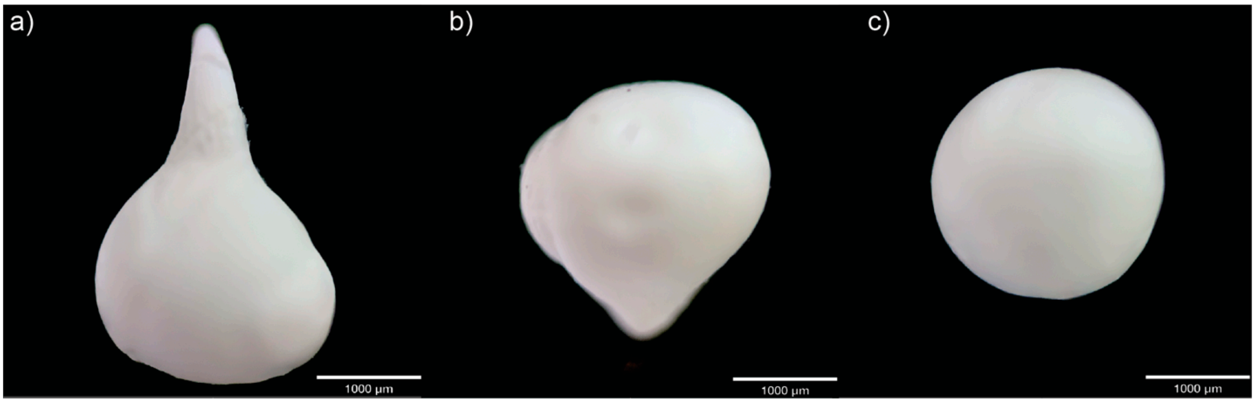

Figure S1. Optical images of freeze-dried (**a**) neat, (**b**) thermo-gel, and (**c**) salt-gel cellulose beads. All scale bars = 1000 µm.

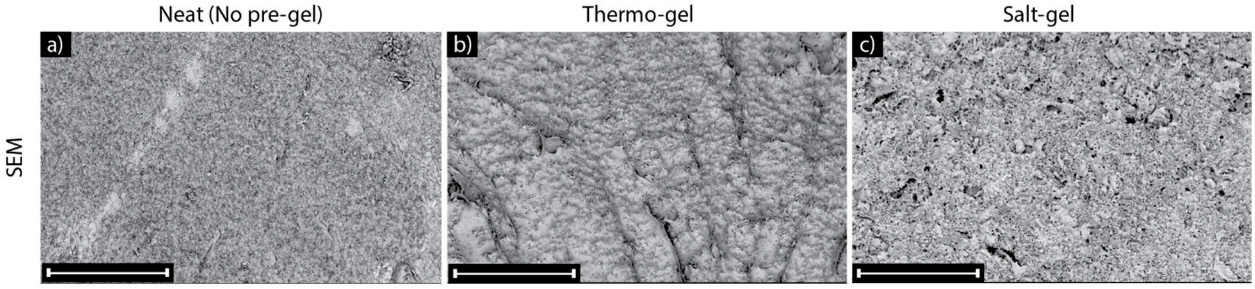

Figure S2. Surface SEM images of (**a**) neat cellulose (no pre-gel), (**b**) thermo-gel, and (**c**) salt-gel beads. SEM revealed that neat and thermo-gel beads had smaller pore architecture that limited diffusion, while salt-gel beads had a larger pore architecture that facilitated diffusion. All scale bars = 80 µm corresponding to 2000× magnification.

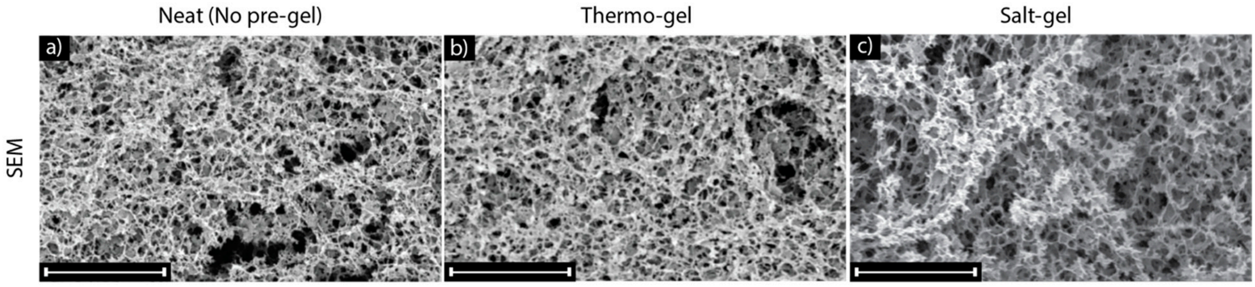

Figure S3. Cross-section SEM images of (**a**) neat cellulose (no pre-gel), (**b**) thermo-gel, and (**c**) salt-gel beads. SEM revealed that neat and thermo-gel beads had smaller pore architecture that limited diffusion, while salt-gel beads had a larger pore architecture that facilitated diffusion. All scale bars = 10 μm corresponding to 15,000× magnification.

Table S1. The electrophoretic mobility and dynamic viscosity were measured for CNF solutions and suspensions as a function of salt concentration.

Salt Concentration (wt%):	Solution/Suspension:	Electrophoretic Mobility (mV):	Dynamic Viscosity (cP):
	CNF Solution – NaOH/Urea	0.03 + 0.00	81.46 + 0.21
	CNF Suspension – DI Water	-2.13 + 0.09	2.02 + 0.09
0 (0 mM)	CNF Suspension – NaOH/Urea	0.55 + 0.27	4.96 + 0.77
	CNF Solution – NaOH/Urea	-0.17 + 0.02	111.16 + 1.98
	CNF Suspension – DI Water	-0.75 + 0.05	2.05 + 0.10
0.25 (56 mM)	CNF Suspension – NaOH/Urea	-0.07 + 0.20	5.80 + 0.24
	CNF Solution – NaOH/Urea	-0.46 + 0.11	127.39 + 1.28
	CNF Suspension – DI Water	-0.50 + 0.02	2.11 + 0.23
0.5 (111 mM)	CNF Suspension – NaOH/Urea	-0.46 + 0.03	5.84 + 0.38
	CNF Solution – NaOH/Urea	-1.00 + 0.08	158.36 + 2.93
	CNF Suspension – DI Water	-0.40 + 0.01	2.19 + 0.26
1 (225 mM)	CNF Suspension – NaOH/Urea	-0.58 + 0.04	6.38 + 0.77
	CNF Solution – NaOH/Urea	-1.57 + 0.15	330.70 + 14.15
	CNF Suspension – DI Water	-0.24 + 0.08	2.38 + 0.21
2 (449 mM)	CNF Suspension – NaOH/Urea	-0.79 + 0.22	6.38 + 0.77

Table S2. The hydrodynamic radius was measured via dynamic light scattering (DLS) as a function of salt concentration.

Salt Concentration:	CNF Solution (nm):	CNF Suspension in NaOH/Urea (nm):	CNF Suspension in DI Water (nm):
2 wt%	12130	10800	7728
1 wt%	11220	6776	7255
0.5 wt%	10630	4922	4768
0.25 wt%	5140	4635	4362
Control	3219	1812	3624

Table S3. EDS Analysis on neat cellulose and salt-gel beads shows that the neutralization procedure washes away the salt, which is a desired result for uses of salt-gel beads for biomedical applications such as oral drug delivery.

Bead Type:	After Fabrication or Neutralization:	Carbon:	Oxygen:	Sodium:	Chlorine:
Salt-gel Bead (Surface)	Fabrication	46.36%	49.19%	1.53%	2.92%
Neat Cellulose Bead (Surface)	After Neutralization	46.74%	53.26%	0.00%	0.00%
Neat Cellulose Bead (Cross-Section)	After Neutralization	45.77%	54.23%	0.00%	0.00%
Salt-gel Bead (Surface)	After Neutralization	47.74%	52.26%	0.00%	0.00%
Salt-gel Bead (Cross-Section)	After Neutralization	49.22%	50.78%	0.00%	0.00%

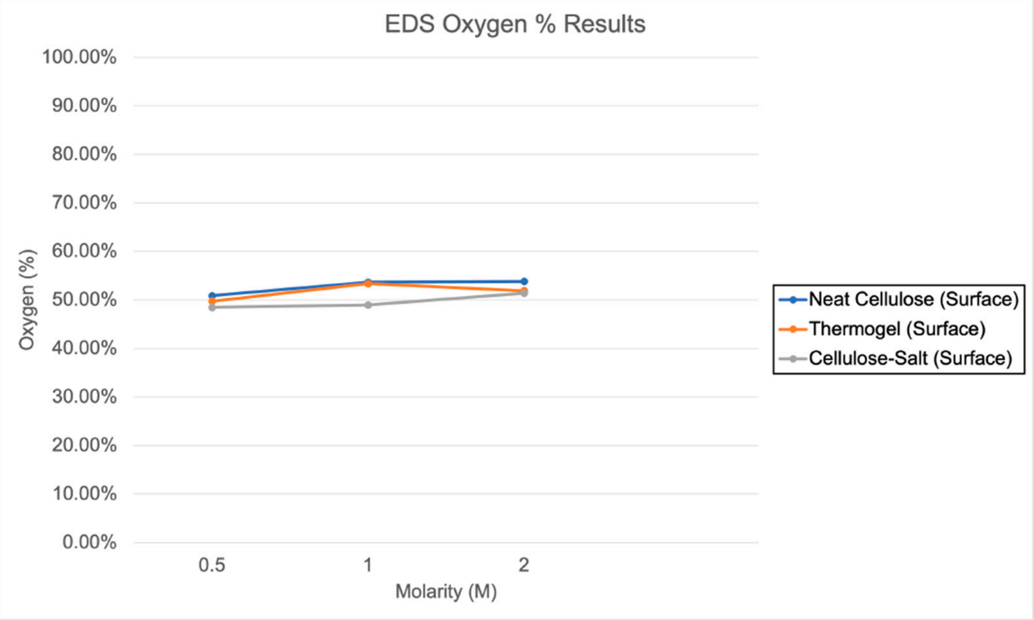

Figure S4. EDS Analysis on neat cellulose, thermo-gel, and salt-gel beads shows little to no change in percent oxygen as the HCl coagulation bath increases from 0.5 to 2 M. Salt-gel beads displayed the lowest percentage of oxygen in each of the different concentrated HCl coagulation baths.

Nano-CT 3-D GIFS: To access 360° rotating GIFS of the Nano-CT reconstructions, visit the following link: https://www.artbiomass.com/research-gallery

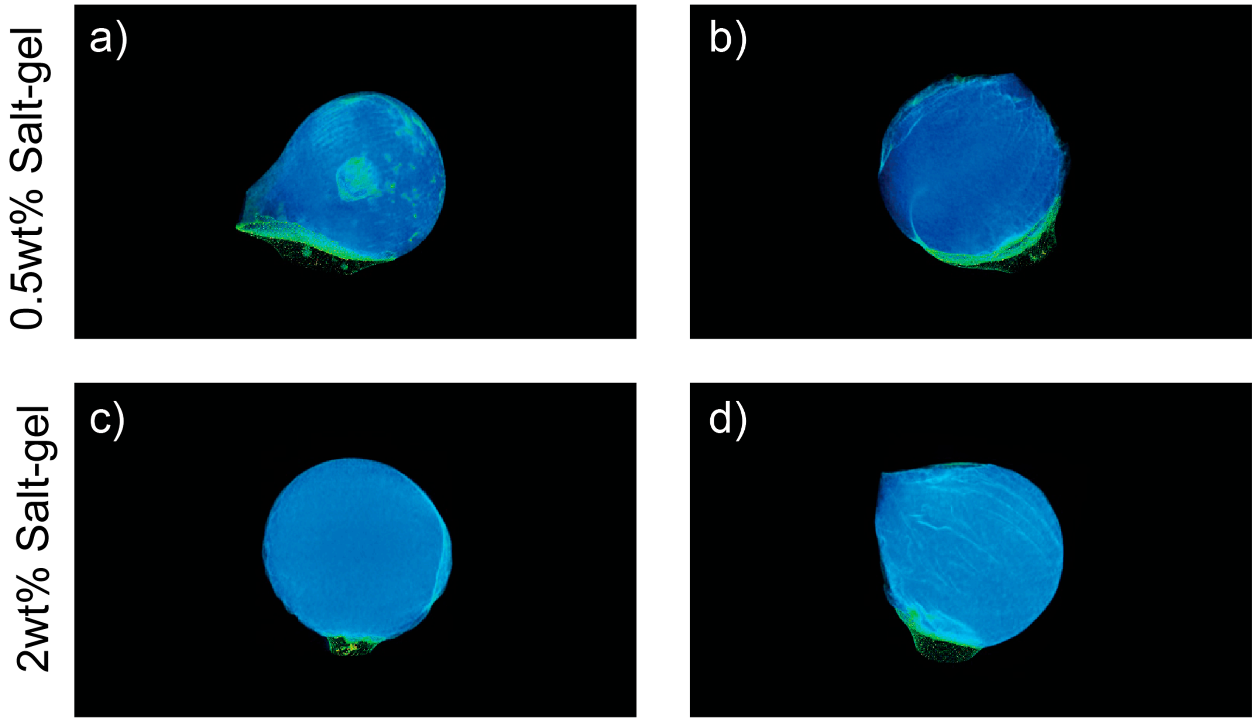

Figure S5. 3-D nano-CT scans were taken of (**a**,**b**) 0.5 wt% salt-gel and 2 wt% salt-gel beads fabricated in a 2M HCl coagulation bath. Nano-CT revealed that increasing the salt concentration improved the uniformity of the internal structure of cellulose beads. (**a**,**b**) 0.5 wt% salt-gel beads displayed both beads with a highly ordered center and uniformity throughout. (**c**,**d**) 2 wt% salt-gel beads displayed uniformity throughout.

Oliver-Pharr model to determine the Young's modulus:

First, the data was fit to a power law expression (Equation S1) using linear regression to determine the fitting parameters,

$$P = B(h - h_f)^m \tag{S1}$$

where P is the force, h is the displacement, B and m are fitting parameters, and h_f is the final unloading displacement. Next, the derivative of Equation S1 was taken to determine the stiffness of the unloading curve (Equation S2),

$$S = \left(\frac{dP}{dh}\right)_{h=h_{max}} = Bm(h - h_f)^{m-1} \tag{S2}$$

where S is the stiffness, h_{max} is the maximum displacement, and B and m are fitting parameters. The calculated stiffness from Equation S2 is then used to determine the contact depth (Equation S3),

$$h_c = h_{max} - \epsilon \frac{P_{max}}{S} \tag{S3}$$

where h_c is the contact depth, ϵ is the punch geometry fitting parameter based on the probe, and P_{max} is the maximum load. Since a spherical ruby tip probe is used, the punch geometry fitting parameter is 0.75. The contact area for a spherical probe is calculated using Equation S4,

$$A_c = \pi(2R_i h_c - h_c^2) \tag{S4}$$

where A_c is the contact area and R_i is the radius of the tip of the probe. The radius of the spherical tip ruby probe used was 125 µm. Next, the reduced elastic modulus is calculated using Equation S5,

$$E_r = \frac{\sqrt{\pi}}{2\beta}\frac{S}{\sqrt{A_c}} \quad \text{(S5)}$$

where E_r is the reduced elastic modulus and β is a fitting parameter based on geometry equal to 1 for spherical tips. Finally, the reduced elastic modulus is used to calculate the Young's modulus (Equation S6),

$$E = E_r(1 - \upsilon^2) \quad \text{(S6)}$$

where E is the Young's modulus and υ is the Poisson's ratio for cellulose is 0.30 [1,2].

Table S4. Swelling/Shrinking Study of Cellulose Beads in a Simulated Gastrointestinal Tract Environment.

Solution and Duration:	Neat-Cellulose Beads Swelling Ratio	Thermo-gel Beads Swelling Ratio	Salt-gel Beads Swelling Ratio
**Water (2 h)**	1.12 + 0.44	1.09 + 0.87	1.10 + 0.53
**SGF (2 h)**	0.77 + 0.52	0.93 + 0.50	0.77 + 0.16
**Water (8 h)**	1.14 + 0.45	1.16 + 0.69	1.14 + 0.43
**SIF (2 h SGF + 6 h SIF)**	1.08 + 0.27	1.02 + 0.68	1.05 + 0.11

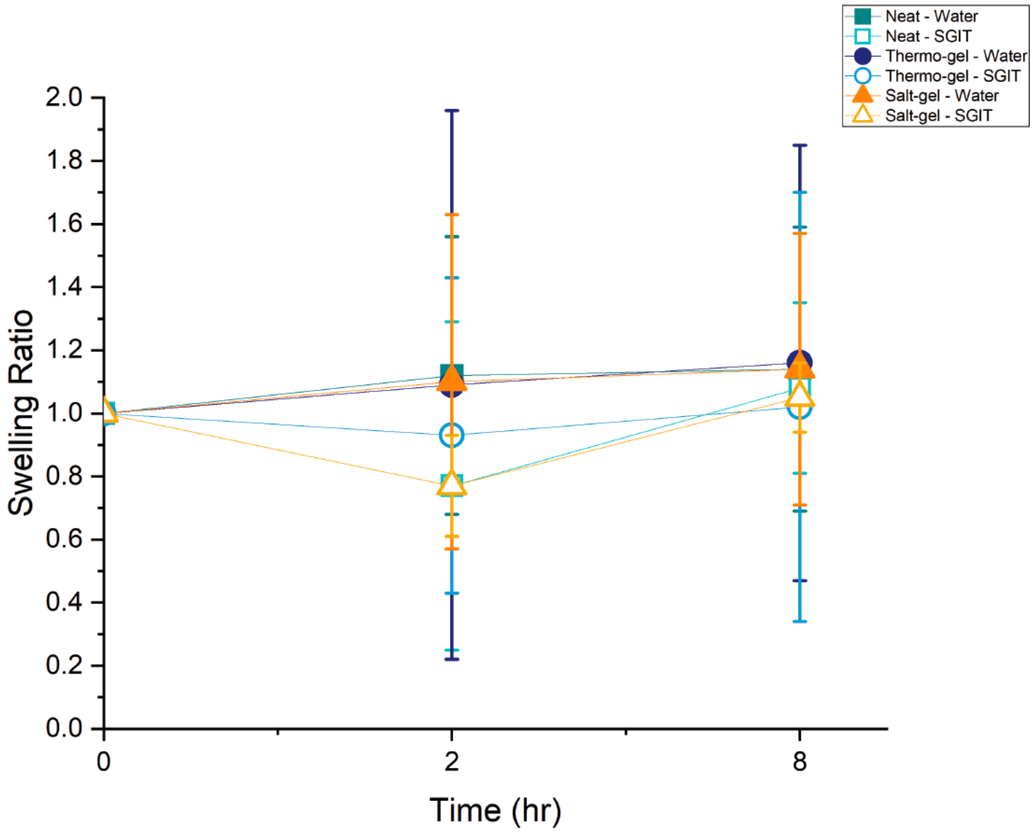

Figure S6. Swelling/Shrinkage Study of Cellulose Beads in a Simulated Gastrointestinal Tract Environment—Neat, thermo-gel, and salt-gel beads were subjected to a control (water), simulated gastric (SGF), and simulated intestinal (SIF) fluid environment and incubated for 2 h (SGF) or 8 h (SIF). The swelling ratio was calculated by the quotient of the final volume by the initial volume.

References

1. Oliver, W.C.; Pharr, G.M.; Introduction, I. An improved technique for determining hardness and elastic modulus using load and displacement sensing indentation experiments. *J. Mater. Res.* **1992**, *7*, 1564–1583.
2. Roberts, R.J.; Rowe, R.C.; York, P. The Poisson's ratio of microcrystalline cellulose. *Int. J. Pharm.* **1994**, *105*, 177–180.

Disclaimer/Publisher's Note: The statements, opinions and data contained in all publications are solely those of the individual author(s) and contributor(s) and not of MDPI and/or the editor(s). MDPI and/or the editor(s) disclaim responsibility for any injury to people or property resulting from any ideas, methods, instructions or products referred to in the content.
